# The prevalence, incidence, management and risks of atrial fibrillation in an elderly Chinese population: a prospective study

**DOI:** 10.1186/s12872-015-0023-3

**Published:** 2015-05-08

**Authors:** Li-Hua Li, Chang-Sheng Sheng, Bang-Chuan Hu, Qi-Fang Huang, Wei-Fang Zeng, Ge-Le Li, Ming Liu, Fang-Fei Wei, Lu Zhang, Yuan-Yuan Kang, Jie Song, Shuai Wang, Yan Li, Shao-Wen Liu, Ji-Guang Wang

**Affiliations:** Centre for Epidemiological Studies and Clinical Trials, Shanghai Key Laboratory of Hypertension, The Shanghai Institute of Hypertension, Ruijin Hospital, Shanghai Jiaotong University School of Medicine, Shanghai, 200025 China; Department of Cardiology, Shanghai First People’s Hospital, Shanghai Jiaotong University School of Medicine, Shanghai, China

**Keywords:** Atrial fibrillation, Epidemiology, Elderly Chinese, Mortality

## Abstract

**Background:**

There is limited information on prevalent and incident atrial fibrillation in Chinese. We aimed to investigate the prevalence, incidence, management and risks of atrial fibrillation in an elderly Chinese population.

**Methods:**

In a population—based prospective study in elderly (≥60 years) Chinese, we performed cardiovascular health examinations including a 12-lead electrocardiogram at baseline in 3,922 participants and biennially during follow-up in 2,017 participants. We collected information on vital status during the whole follow-up period.

**Results:**

The baseline prevalence of atrial fibrillation was 2.0 % (*n* = 34) in 1718 men and 1.6 % (*n* = 36) in 2204 women. During a median 3.8 years of follow-up, the incidence rate of atrial fibrillation (*n* = 34) was 4.9 per 1000 person-years (95 % confidence interval [CI], 3.4–6.9). In univariate analysis, both the prevalence and incidence of atrial fibrillation were higher with age advancing (*P* < 0.0001) and in the presence of coronary heart disease (*P* ≤ 0.02). Of the 104 prevalent and incident cases of atrial fibrillation, only 1 (1.0 %) received anticoagulant therapy (warfarin). These patients with atrial fibrillation, compared with those with sinus rhythm, had significantly higher risks of all-cause (*n* = 261, hazard ratio [HR] 1.87, 95 % CI, 1.09–3.20, *P* = 0.02), cardiovascular (*n* = 136, HR 3.78, 95 % CI 2.17–6.58, *P* < 0.0001) and stroke mortality (*n* = 44, HR 6.31, 95 % CI 2.81–14.19, *P* = 0.0003).

**Conclusions:**

Atrial fibrillation was relatively frequent in elderly Chinese, poorly managed and associated with higher risks of mortality.

## Background

Atrial fibrillation is a common cardiac arrhythmia, can be symptomatic with tachycardia, but is usually asymptomatic or mildly symptomatic. Atrial fibrillation can be permanent, but in the beginning is often paroxysmal. That explains why the detection rate of atrial fibrillation is low in the clinical practice and why the prevalence of atrial fibrillation varies between studies. Nonetheless, it is known that the prevalence and incidence of atrial fibrillation increase with ageing [1–4] and in the presence of several cardiovascular risk factors and diseases [2–5]. In China, with the substantial decrease in the prevalence of rheumatic valvular heart disease, and substantial increase in the prevalence of hypertension [6] and coronary heart disease [7] in the past two decades, the latter diseases have become the major cause of atrial fibrillation.

Atrial fibrillation is one of the major causes of embolic stroke, and is associated with higher risks of cardiovascular events and mortality [8, 9]. Some atrial fibrillation, especially in the paroxysmal type or phase, can be pharmaceutically or electrically converted to normal heart rhythm, and some atrial fibrillation can be prevented for recurrence with medications or by surgery or catheter—based ablation. However, many or most of atrial fibrillation, require chronic pharmacological management for the prevention of cardiovascular complications, such as, the use of warfarin or other anticoagulants for the prevention of embolic stroke.

At present, there is still very limited information on the prevalence of atrial fibrillation in China, and to the best of our knowledge no prospective data on the incidence, management and risks of atrial fibrillation. We recently performed biennial recordings of electrocardiogram (ECG) in an elderly Chinese population. In the present study, we investigated the prevalence, incidence, management and risks of atrial fibrillation in this elderly population.

## Methods

### Study population

Our study was conducted in the framework of the Chronic Disease Detection and Management in the Elderly (≥60 years) Program supported by the municipal government of Shanghai [10, 11]. In a newly urbanized suburban town, 30 km from the city center, we invited all residents of 60 years or older to take part in comprehensive examinations of cardiovascular disease and risk. We adhered to the principles of the Declaration of Helsinki, and the study protocol was approved by the Ethics Committee of Ruijin Hospital, Shanghai Jiaotong University School of Medicine. All participants gave written informed consent.

A total of 4,750 participants (participation rate 90 %) were enrolled from 2006 to 2011, and followed up for clinical and biochemical examinations biennially and for vital status and cause of death till September 1, 2011. We excluded 828 participants from the present analysis, because ECG was not performed (*n* = 694) or because of missing other information (*n* = 134). Thus, the number of participants included in the present analysis was 3,922.

### Rest 12-lead ECG

Rest ECG (ECG-9130P, Nihon Kohden, Japan) was performed at baseline and during follow-up by a physician. ECGs were read by two cardiologists for the determination of atrial fibrillation, defined according to the 2011 ACC/AHA/ESC guidelines [12]. Atrial flutter and atrial tachycardia were also recorded, but only atrial fibrillation was included in the present analysis. The prevalent case was the presence of atrial fibrillation at baseline, and the incident case was the absence of atrial fibrillation at baseline but the presence of atrial fibrillation on at least one of the ECG recordings during follow-up. Patients with atrial fibrillation were required to provide information on the use of oral anticoagulants. We also estimated the risk of stroke in patients with atrial fibrillation using the CHADS_2_ scoring system [13].

### Other field work

One experienced physician measured each participant’s blood pressure three times consecutively using a validated oscillometric blood pressure monitor (Omron 7051, Kyoto, Japan), after the subjects had rested for at least 5 min in the sitting position. The same observer also administered a standardized questionnaire to collect information on medical history, lifestyle and use of medications. Hypertension was defined as a sitting blood pressure (average of three readings) of at least 140 mmHg systolic or 90 mmHg diastolic, or as the use of antihypertensive drugs. A trained technician performed anthropometric measurements. Body mass index was the body weight in kilograms divided by the body height in meters squared. Obesity was defined as a body mass index of at least 30 kg/m^2^. Coronary heart disease included self-reported history of myocardial infarction, angina pectoris or coronary revascularizations. Valvular heart disease included self-reported history of rheumatic or degenerative valvular heart disease.

Venous blood samples were drawn after overnight fasting for the measurement of plasma glucose concentration and serum concentrations of total cholesterol and triglycerides. Diabetes mellitus was defined as plasma glucose of at least 7.0 mmol/l fasting or 11.1 mmol/l at any time, or as the use of anti-diabetic agents. Serum creatinine was measured by the Jaffe kinetic method. Estimated glomerular filtration rate (eGFR) was calculated using the Modification of Diet in Renal Disease (MDRD) Study equation: GFR = 186 × (serum creatinine, mg/dl)^−1.154^ × (age, years)^−0.203^ × (0.742 if female) [14]. Chronic kidney disease (CKD) was defined as eGFR < 60 ml/min × 1.73m^2^.

### Follow-up

All participants alive were invited to participate in the biennial comprehensive examinations of cardiovascular disease and risk. Information on vital status and the cause of death was obtained from the official death certificate, with further confirmation by the local Community Health Center and family members of the deceased people. ICD-9 (International Classification of Diseases, 9th Revision) was used to classify the cause of death. Cardiovascular mortality included deaths attributable to stroke, heart failure, myocardial infarction, and other cardiovascular diseases.

### Statistical analysis

For database management and statistical analysis, we used SAS software (version 9.2, SAS Institute, Cary, NC, USA). We calculated the prevalence of atrial fibrillation according to the data collected at baseline in the period from 2006 to 2011. We calculated the incidence of atrial fibrillation according to the biennial ECG examinations in the subjects enrolled in 2009 or earlier and the incidence of fatal events in all subjects. The 95 % binomial confidence intervals of the prevalence and incidence rates were computed according to the exact method [15]. Means and proportions were compared with the Student’s *t*-test and *χ*^2^ test, respectively. Logistic and Cox regression analyses were performed to compute odds ratios and hazard ratios, respectively, with their 95 % confidence intervals. The log-rank test was used to compare the cumulative incidence of all-cause, cardiovascular and stroke mortality between patients with atrial fibrillation and those without. Kaplan–Meier survival function was used to show the time to death. All *P* values were two-sided, and significance was defined as a *P* ≤ 0.05.

## Results

### Characteristics of the study population

At baseline, the 3,922 participants included 2,204 women (56.2 %) and 2,344 hypertensive patients (59.8 %), of whom 1,593 patients (68.0 %) took antihypertensive medication. Table 1 shows the baseline characteristics of participants by sex.

**Table 1:** Baseline characteristics of the study population

Characteristic	Men	Women	*P*
	(*n* = 1718)	(*n* = 2204)	
Age, years	68.7 ± 7.1	69.5 ± 7.4	0.002
Body mass index, kg/m^2^	23.5 ± 3.5	23.8 ± 3.7	0.006
Systolic blood pressure, mm Hg	137.4 ± 19.9	138.2 ± 19.7	0.23
Diastolic blood pressure, mm Hg	80.3 ± 10.9	78.7 ± 10.5	<0.0001
Pulse rate, beats/min	74.6 ± 12.0	77.4 ± 11.7	<0.0001
Serum total cholesterol, mmol/l	5.3 ± 1.5	5.5 ± 1.5	<0.0001
Serum triglycerides, mmol/l	1.5 ± 0.8	1.7 ± 0.8	<0.0001
Serum creatinine, μmol/l	90.5 ± 31.3	86.0 ± 27.5	<0.0001
Fasting plasma glucose, mmol/l	5.2 ± 1.2	5.4 ± 1.3	<0.0001
Current smoking, *n* (%)	909 (52.9)	48 (2.2)	<0.0001
Alcohol intake, *n* (%)	627 (36.5)	30 (1.4)	<0.0001
Hypertension, *n* (%)	1031 (60.0)	1313 (59.6)	0.78
Use of antihypertensive drugs, n (%)	685 (39.9)	908 (41.2)	0.40
Diabetes mellitus, *n* (%)	116 (6.8)	183 (8.3)	0.07
Antidiabetic treatment, *n* (%)	61 (3.6)	91 (4.1)	0.35
History of cardiovascular disease			
Coronary heart disease, *n* (%)	10 (0.6)	6 (0.3)	0.13
Valvular heart disease, *n* (%)	1 (0.1)	0 (0)	0.26
Prevalence of atrial fibrillation, *n* (%)	34 (2.0)	36 (1.6)	0.42
CHADS_2_ score ≥1 point, *n* (%)	1191 (69.3)	1531 (69.5)	0.92

Values are mean ± SD, or number (%) of subjects. For definitions of hypertension, diabetes mellitus, coronary heart disease, valvular heart disease, atrial fibrillation, and CHADS_2_ score, see Methods

### Prevalence and incidence of atrial fibrillation

**Fig. 1:**
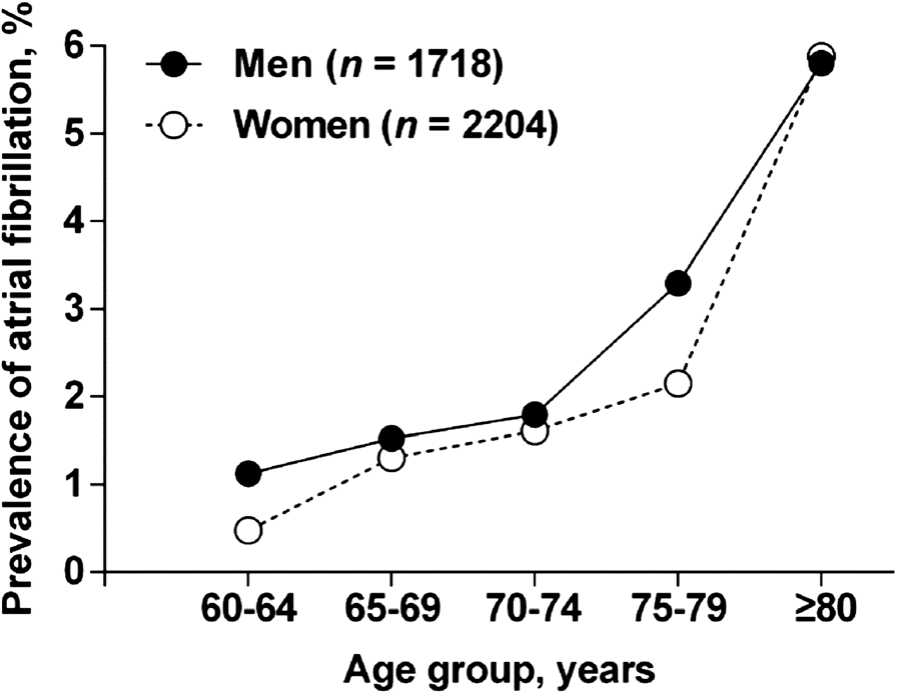
Prevalence of atrial fibrillation by sex and age. Solid and open symbols represent men and women, respectively

The baseline prevalence of atrial fibrillation was 1.8 % (*n* = 70), was slightly higher in men than women (2.0 % *vs.* 1.6 %, *P* = 0.42), and increased significantly with advancing age (Table 2 and Fig. 1). During a median follow-up of 3.8 years, 34 of the 2,017 participants who did not have atrial fibrillation at baseline had atrial fibrillation on at least one ECG recording during follow-up. Overall, the incidence rate of atrial fibrillation was 4.9 per 1,000 person—years (95 % confidence interval [CI], 3.4–6.9, Table 2).

**Table 2:** Prevalence and incidence of atrial fibrillation by age group

Age group	Prevalence (*n* = 3922)					Incidence (*n* = 2017)				
	Number of participants	Number of AF cases	Rate (%)	95 % CI		Number of person-years	Number of AF cases	Rate (per 1000 person-years)	95 % CI	
				Lower	Upper				Lower	Upper
60–64	1550	12	0.8	0.4	1.4	2297	6	2.6	1.0	5.7
65–69	708	10	1.4	0.7	2.6	1429	3	2.1	0.4	6.1
70–74	767	13	1.7	0.9	2.9	1619	11	6.8	3.4	12.1
75–79	538	14	2.6	1.4	4.3	1116	11	9.9	4.9	17.6
≥80	359	21	5.9	3.7	8.8	464	3	6.5	1.3	18.8
Total	3922	70	1.8	1.4	2.3	6925	34	4.9	3.4	6.9

AF indicates atrial fibrillation. 95 % confidence intervals (CI) were computed according to binominal distribution

### Risk factors of prevalent and incident atrial fibrillation

In univariate logistic regression analyses, the baseline prevalence of atrial fibrillation was significantly higher in participants of at least 70 years old versus those of 60–69 years (*n* = 1664 *vs. n* = 2258, odds ratio [OR] 3.02, 95 % CI 1.82–5.02, *P* <0.0001, Table 3) and in patients with coronary heart disease (*n* = 16, OR 8.06, 95 % CI 1.80–36.17, *P* = 0.006). After adjustment for age, this association became non-significant for coronary heart disease (*P* = 0.14). In further analyses, we found significant (*P* = 0.0007) interaction between hypertension and age in relation to the prevalence of atrial fibrillation. Indeed, the association between the prevalence of atrial fibrillation and hypertension was positive in participants younger than 80 years (*n* = 3563, OR 2.21, 95 % CI 1.12–4.37, *P* = 0.02) but negative in those of 80 years and older (*n* = 359, OR 0.27, 95 % CI 0.11–0.69, *P* = 0.006).

**Table 3:** Association of prevalent and incident atrial fibrillation with cardiovascular risk factors in univariate cross-sectional and prospective analyses respectively

Variable	Cross-sectional		Prospective	
	OR (95 % CI)	*P*	HR (95 % CI)	*P*
Age (≥70 *vs.* 60–69 years)	3.02 (1.82–5.02)	<0.0001	4.47 (1.98–10.06)	<0.0001
Obesity	0.63 (0.15–2.57)	0.52	1.47 (0.35–6.12)	0.60
Current smoking	1.48 (0.82–2.69)	0.20	1.28 (0.56–2.91)	0.55
Alcohol intake	0.95 (0.54–1.66)	0.84	1.27 (0.50–3.19)	0.60
Hypertension	1.30 (0.79–2.13)	0.31	1.33 (0.66–2.65)	0.42
Diabetes mellitus	0.54 (0.17–1.72)	0.30	0.79 (0.19–3.30)	0.74
Coronary heart disease	8.06 (1.80–36.17)	0.006	9.10 (2.18–38.0)	0.02
Chronic kidney disease	1.32 (0.81–2.14)	0.26	0.70 (0.34–1.42)	0.31

For definitions of obesity, hypertension, diabetes mellitus, coronary heart disease, and chronic kidney disease, see Methods

**Fig. 2:**
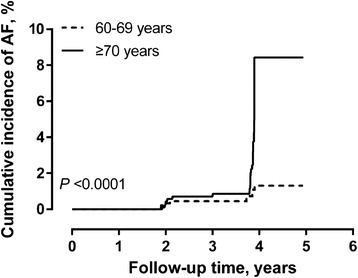
Kaplan-Meier survival curve for incident atrial fibrillation. The analysis was stratified by age group (≥70 *vs.* 60–69 years). A *P* value by Log-rank test was given for the comparison

The univariate Cox regression analysis produced confirmatory results and identified similar risk factors of the incident atrial fibrillation as in the cross-sectional analysis (Table 3 and Fig. 2). Nonetheless, the association remained statistically significant in age—adjusted analysis for coronary heart disease (*P* = 0.0006), and the interaction between hypertension and age in relation to the incidence of atrial fibrillation was borderline significant (*P* = 0.06).

### Management of atrial fibrillation

Of the 104 prevalent (*n* = 70) and incident (*n* = 34) cases of atrial fibrillation, 90 had a CHADS2 score of at least 1 point and hence were indicated for anticoagulant therapy. However, overall, only 8 (7.7 %) were aware of the disease, 5 (4.8 %) received antiplatelet treatment (aspirin), 1 (1.0 %) received anticoagulant therapy (warfarin), and 2 (1.9 %) had a history of stroke. In addition, none of the patients with atrial fibrillation used β-blockers, 7 (6.7 %) had a heart rate faster than 110 beats per minute, and the mean heart rate was 85 beats per minute (range: 56 to 121).

### Association of atrial fibrillation with mortality

**Fig. 3:**
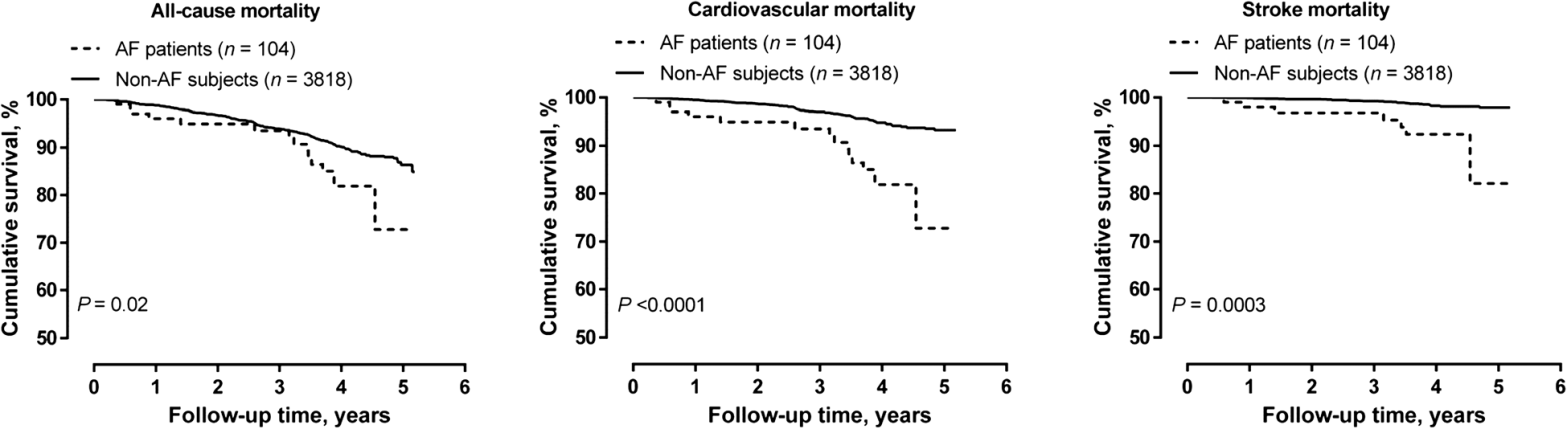
Kaplan-Meier survival curves for all-cause, cardiovascular and stroke mortality. The analysis for all-cause (left panel), cardiovascular (middle panel), and stroke (right panel) mortality were stratified by the diagnosis of atrial fibrillation (presence *vs.* absence of atrial fibrillation). A *P* value by Log-rank test was given for each comparison

During follow-up, the cumulated number of person—years was 13,727, and the number of all-cause, cardiovascular, and stroke deaths was 261, 136 and 44, respectively. In unadjusted Cox regression analyses, the 104 patients with atrial fibrillation, compared with those with sinus rhythm (*n* = 3818), had significantly higher risk of all-cause (*n* = 261, hazard ratio [HR] 1.87, 95 % CI, 1.09–3.20, *P* = 0.02), cardiovascular (*n* = 136, HR 3.78, 95 % CI 2.17–6.58, *P* <0.0001) and stroke mortality (*n* = 44, HR 6.31, 95 % CI 2.81–14.19, *P* = 0.0003, Fig. 3). After adjustment for sex, age, body mass index, current smoking, alcohol intake and hypertension, the corresponding hazard ratios were 1.13 (95 % CI 0.66–1.95, *P* = 0.65), 2.16 (95 % CI 1.23–3.80, *P* = 0.007), and 3.86 (95 % CI 1.69–3.85, *P* = 0.001), respectively.

## Discussion

Our main finding was that the overall prevalence and incidence in our elderly Chinese population were 1.8 % and 4.9 per 1,000 person—years, respectively, and increased with advancing age and in the presence of coronary heart disease. The management of atrial fibrillation was poor, with low awareness, under-use or no use of proven treatment, and high risks of mortality.

Our results on the prevalence of atrial fibrillation are similar to the data of most [4, 16], though not all [17], recent epidemiological studies in China. In 29,079 participants recruited from 13 provinces across China in 2004, the overall crude prevalence of atrial fibrillation in elderly participants (≥60 years) was 2.0 %, which was higher in men (2.3 %) than in women (1.7 %) [4]. Their algorithm for case ascertainment of atrial fibrillation was based on 12-lead ECG, medical history, or prior ECG records for episode of atrial fibrillation, which might be more likely to identify those with paroxysmal atrial fibrillation and therefore yielded slightly higher prevalence of atrial fibrillation than our study. In a similar study in elderly Chinese (≥60 years) using ECG and medical history, the crude prevalence of atrial fibrillation was 1.83 % in men as well as women [16]. However, in the Guangzhou Biobank Cohort Study [17] that used similar diagnostic technique, the prevalence of atrial fibrillation was only 1.04 % in elderly participants (≥60 years), much lower than the present study and the abovementioned multicenter studies. In addition, if our study would be compared with studies of other populations in Asia and other parts of the world, the prevalence of atrial fibrillation was similar among Eastern Asians [3, 18, 19], but significantly higher in American [20, 21] and European populations [22, 23].

To the best of our knowledge, the present study was the first that reported the incidence of atrial fibrillation in elderly Chinese. The incidence of atrial fibrillation in our study was lower than that was reported in 30,010 Japanese participants (4.8 *vs.* 9.3 per 1,000 person—years) [24] and in several American [25, 26] and European populations [23, 27], such as the elderly Germans [23, 27], US Medicare beneficiaries [25], and older adults in the Cardiovascular Health Study [26]. Inter-ethnic difference in the prevalence and incidence of atrial fibrillation has been reported previously [26, 28]. In addition to the difference in genetic architecture, lifestyle and cardiovascular risk and disease pattern, the frequency of ECG recordings might be a key factor that influences the identification and hence the incidence of atrial fibrillation.

Our finding on the interaction between age and hypertension in relation to the prevalence of atrial fibrillation remains incompletely understood. Nevertheless, we believe that selective survival might explain the inverse association between hypertension and the prevalence of atrial fibrillation in subjects older than 80 years. The combination of hypertension and atrial fibrillation makes a person less likely survive to an age of 80 years or older. In addition, the treatment and control (<140/90 mmHg) rate of hypertension were 68.0 % and 27.0 %. The relatively high treatment and control rate of hypertension might diminish the power of our study to show a positive association between hypertension and the risk of atrial fibrillation.

An astonishing finding of our study is the low—use of anticoagulant therapy in a community in the suburb of Shanghai. Anticoagulant therapy is well proven and strongly recommended for the prevention of stroke in patients with atrial fibrillation [29]. However, only 1 (1.0 %) of the 104 patients with atrial fibrillation received warfarin, while 5 patients received antiplatelet treatment, aspirin. Previous studies in China also reported under—use of anticoagulant therapy in patients with atrial fibrillation from 2.7 % [4] to 9.27 % [30]. The extremely low use in our study suggests that low awareness might be one of the major barriers for the use of proven therapy in these very high-risk patients. The poor management should be the main reason why patients with atrial fibrillation had such a high risk of mortality as observed in our prospective study.

Our study should be interpreted within the context of its strength and limitations. Our study was the first population—based study that reported the prevalence, incidence, management, and risks of atrial fibrillation in elderly Chinese. However, our study was conducted in a single community, which renders our results less representative than a multicenter study. Our study had relatively small sample size and short follow-up and hence small number of prevalent and incident cases of atrial fibrillation. We did not perform echocardiography and hence could not accurately assess valvular heart disease and left ventricular dysfunction and their relationship with atrial fibrillation. Coronary artery disease was self-reported, and hence might be substantially underestimated. Our study might be inadequately powered to reveal association between coronary artery disease and incident atrial fibrillation. With the currently available data, we were unable to differentiate types of atrial fibrillation. Finally, the ascertainment of atrial fibrillation was based on biennial rest ECGs only, which might lead to under-detection, and hence under-estimation of the disease risk and burden.

## Conclusions

Atrial fibrillation was relatively frequent in elderly Chinese, poorly managed and associated with higher risks of mortality. Further research is required to investigate whether increasing frequency of ECG tracing would improve awareness and management of atrial fibrillation in Chinese, particularly the use of well-proven therapeutic treatment, such as warfarin or other anticoagulants.

## References

[1] Feinberg WM, Blackshear JL, Laupacis A, Kronmal R, Hart RG (1995). Prevalence, age distribution, and gender of patients with atrial fibrillation: analysis and implications. Arch Intern Med.

[2] Psaty BM, Manolio TA, Kuller LH, Kronmal RA, Cushman M, Fried LP, White R, Furberg CD, Rautaharju PM (1997). Incidence of and risk factors for atrial fibrillation in older adults. Circulation.

[3] Jeong JH (2005). Prevalence of and risk factors for atrial fibrillation in Korean adults older than 40 years. J Korean Med Sci.

[4] Zhou Z, Hu D (2008). An epidemiological study on the prevalence of atrial fibrillation in the Chinese population of mainland China. J Epidemiol.

[5] Benjamin EJ, Levy D, Vaziri SM, D'Agostino RB, Belanger AJ, Wolf PA (1994). Independent risk factors for atrial fibrillation in a population—based cohort: the Framingham Heart Study. JAMA.

[6] Wu Y, Huxley R, Li L, Anna V, Xie G, Yao C, Woodward M, Li X, Chalmers J, Gao R (2008). Prevalence, awareness, treatment, and control of hypertension in China: data from the China National Nutrition and Health Survey 2002. Circulation.

[7] Zhang XH, Lu ZL, Liu L (2008). Coronary heart disease in China. Heart.

[8] Wolf PA, Abbott RD, Kannel WB (1991). Atrial fibrillation as an independent risk factor for stroke: the Framingham Study. Stroke.

[9] Benjamin EJ, Wolf PA, D'Agostino RB, Silbershatz H, Kannel WB, Levy D (1998). Impact of atrial fibrillation on the risk of death: the Framingham Heart Study. Circulation.

[10] Sheng CS, Liu M, Kang YY, Wei FF, Zhang L, Li GL, Dong Q, Huang QF, Li Y, Wang JG (2013). Prevalence, awareness, treatment and control of hypertension in elderly Chinese. Hypertens Res.

[11] Sheng CS, Liu M, Zeng WF, Huang QF, Li Y, Wang JG (2013). Four—limb blood pressure as predictors of mortality in elderly Chinese. Hypertension.

[12] Fuster V, Rydén LE, Cannom DS, Crijns HJ, Curtis AB, Ellenbogen KA, Halperin JL, Kay GN, Le Huezey J-Y, Lowe JE (2011). ACCF/AHA/HRS focused updates incorporated into the ACC/AHA/ESC 2006 guidelines for the management of patients with atrial fibrillation: a report of the American College of Cardiology Foundation/American Heart Association Task Force on Practice Guidelines. Circulation.

[13] Gage BF, Waterman AD, Shannon W, Boechler M, Rich MW, Radford MJ (2001). Validation of clinical classification schemes for predicting stroke: results from the National Registry of Atrial Fibrillation. JAMA.

[14] Levey AS, Coresh J, Balk E, Kausz AT, Levin A, Steffes MW, Hogg RJ, Perrone RD, Lau J, Eknoyan G (2003). National Kidney Foundation practice guidelines for chronic kidney disease: evaluation, classification, and stratification. Ann Intern Med.

[15] Clopper C, Pearson ES (1934). The use of confidence or fiducial limits illustrated in the case of the binomial. Biometrika.

[16] Li Y, Wu YF, Chen KP, Li X, Zhang X, Xie GQ, Wang FZ, Zhang S (2013). Prevalence of atrial fibrillation in China and its risk factors. Biomed Environ Sci.

[17] Long MJ, Jiang CQ, Lam TH, Xu L, Zhang WS, Lin JM, Ou JP, Cheng KK (2011). Atrial fibrillation and obesity among older Chinese: the Guangzhou Biobank Cohort Study. Int J Cardiol.

[18] Inoue H, Fujiki A, Origasa H, Ogawa S, Okumura K, Kubota I, Aizawa Y, Yamashita T, Atarashi H, Horie M (2009). Prevalence of atrial fibrillation in the general population of Japan: an analysis based on periodic health examination. Int J Cardiol.

[19] Chien KL, Su TC, Hsu HC, Chang WT, Chen PC, Chen MF, Lee YT (2010). Atrial fibrillation prevalence, incidence and risk of stroke and all-cause death among Chinese. Int J Cardiol.

[20] Ryder KM, Benjamin EJ (1999). Epidemiology and significance of atrial fibrillation. Am J Cardiol.

[21] Go AS, Hylek EM, Phillips KA, Chang Y, Henault LE, Selby JV, Singer DE (2001). Prevalence of diagnosed atrial fibrillation in adults: national implications for rhythm management and stroke prevention: the AnTicoagulation and Risk Factors in Atrial Fibrillation (ATRIA) Study. JAMA.

[22] Andersson P, Londahl M, Abdon NJ, Terent A (2012). The prevalence of atrial fibrillation in a geographically well-defined population in northern Sweden: implications for anticoagulation prophylaxis. J Intern Med.

[23] Wilke T, Groth A, Mueller S, Pfannkuche M, Verheyen F, Linder R, Maywald U, Bauersachs R, Breithardt G (2013). Incidence and prevalence of atrial fibrillation: an analysis based on 8.3 million patients. Europace.

[24] Iguchi Y, Kimura K, Shibazaki K, Aoki J, Kobayashi K, Sakai K, Sakamoto Y (2010). Annual incidence of atrial fibrillation and related factors in adults. Am J Cardiol.

[25] Piccini JP, Hammill BG, Sinner MF, Jensen PN, Hernandez AF, Heckbert SR, Benjamin EJ, Curtis LH (2012). Incidence and prevalence of atrial fibrillation and associated mortality among Medicare beneficiaries: 1993–2007. Circ Cardiovasc Qual Outcomes.

[26] Jensen PN, Thacker EL, Dublin S, Psaty BM, Heckbert SR (2013). Racial differences in the incidence of and risk factors for atrial fibrillation in older adults: the Cardiovascular Health Study. J Am Geriatr Soc.

[27] Ohlmeier C, Mikolajczyk R, Haverkamp W, Garbe E (2013). Incidence, prevalence, and antithrombotic management of atrial fibrillation in elderly Germans. Europace.

[28] Borzecki AM, Bridgers DK, Liebschutz JM, Kader B, Kazis LE, Berlowitz DR (2008). Racial differences in the prevalence of atrial fibrillation among males. J Natl Med Assoc.

[29] Camm AJ, Kirchhof P, Lip GY, Schotten U, Savelieva I, Ernst S, Van Gelder IC, Al-Attar N, Hindricks G, Prendergast B (2010). Guidelines for the management of atrial fibrillation: the Task Force for the Management of Atrial Fibrillation of the European Society of Cardiology (ESC). Eur Heart J.

[30] Sun Y, Hu D, Li K, Zhou Z (2009). Predictors of stroke risk in native Chinese with nonrheumatic atrial fibrillation: retrospective investigation of hospitalized patients. Clin Cardiol.

